# Impact of Helicopter Vibrations on In-Ear PPG Monitoring for Vital Signs—Mountain Rescue Technology Study (MoReTech)

**DOI:** 10.3390/s26010324

**Published:** 2026-01-04

**Authors:** Aaron Benkert, Jakob Bludau, Lukas Boborzi, Stephan Prueckner, Roman Schniepp

**Affiliations:** 1 Department of Neurology, LMU University Hospital, LMU Munich, 81377 Munich, Germany; 2 Institut für Notfallmedizin und Medizinmanagement (INM), LMU University Hospital, LMU Munich, 80336 Munich, Germany; 3 Institute for Rotorcraft and Vertical Flight, Technical University of Munich, 85748 Garching, Germany

**Keywords:** photoplethysmography (PPG), pulse oximetry, motion artifacts, in-ear, helicopter

## Abstract

Pulsoximeters are widely used in the medical care of preclinical patients to evaluate the cardiorespiratory status and monitor basic vital signs, such as pulse rate (PR) and oxygen saturation (SpO_2_). In many preclinical situations, air transport of the patient by helicopter is necessary. Conventional pulse oximeters, mostly used on the patient’s finger, are prone to motion artifacts during transportation. Therefore, this study aims to determine whether simulated helicopter vibration has an impact on the photoplethysmogram (PPG) derived from an in-ear sensor at the external ear canal and whether the vibration influences the calculation of vital signs PR and SpO_2_. The in-ear PPG signals of 17 participants were measured at rest and under exposure to vibration generated by a helicopter simulator. Several signal quality indicators (SQI), including perfusion index, skewness, entropy, kurtosis, omega, quality index, and valid pulse detection, were extracted from the in-ear PPG recordings during rest and vibration. An intra-subject comparison was performed to evaluate signal quality changes under exposure to vibration. The analysis revealed no significant difference in any SQI between vibration and rest (all *p* > 0.05). Furthermore, the vital signs PR and SpO_2_ calculated using the in-ear PPG signal were compared to reference measurements by a clinical monitoring system (ECG and SpO_2_ finger sensor). The results for the PR showed substantial agreement (${\mathrm{CCC}}_{rest}$ = 0.96; ${\mathrm{CCC}}_{vibration}$ = 0.96) and poor agreement for SpO_2_ (${\mathrm{CCC}}_{rest}$ = 0.41; ${\mathrm{CCC}}_{vibration}$ = 0.19). The results of our study indicate that simulated helicopter vibration had no significant impact on the calculation of the SQIs, and the calculation of vital signs PR and SpO_2_ did not differ between rest and vibration conditions.

## 1. Introduction

Pulse oximeters are particularly common in the preclinical setting for monitoring vital parameters such as pulse rate (PR) and oxygen saturation (SpO_2_). Due to their ease of use and the ability to continuously monitor patients, pulse oximetry has become standard practice in monitoring nearly every patient. The functionality of pulse oximetry is based on physical principles such as light reflection and absorption of red (R) and infrared light (IR) [1]. The optically registered signal (Photoplethysmogram, PPG) is divided into two components to calculate the oxygen saturation. The first component is called the direct current component (DC) and is formed by the non-pulsatile absorption of the light signal from the skin, bones, connective tissue, and venous blood [1]. It is influenced by factors like respiration, sympathetic nervous system activity, and thermoregulation [2]. The second component is called alternating current (AC) and varies wave-like due to the increase in arterial blood volume during systolic ejection [1]. PPG signals are prone to errors by artifacts, both affecting baseline shift (DC) and changes in the contour of the pulsatile waveform (AC) [3]. Studies have shown that motion artifacts, especially during physical activities, make it challenging to extract accurate cardiovascular parameters like PR and SpO_2_ [4,5]. These artifacts can mimic actual pulsations, leading to false readings and waveform distortions [6]. Various methods have been proposed to eliminate motion artifacts from PPG signals to improve measurement accuracy and to enhance the quality of vital sign monitoring [4,7,8,9,10]. These algorithms leverage spectral analysis and adaptive filtering techniques to identify and remove frequency components associated with motion artifacts [11]. Additionally, multiple sensors and advanced filtering methods have been explored to mitigate the effects of motion artifacts on PPG signals [12,13]. Patient transportation might also significantly impact PPG-based vital sign monitoring through movements and vibrations from vehicles or helicopters. Artifacts induced by transportation might vary substantially in amplitude, frequency, or degree of freedom from those caused by physical activity. Thus, quality management of PPG-based patient monitoring is fundamental in prehospital patient transportation.

It is known that the anatomical site of PPG extraction influences the vulnerability of the signal to movement artifacts [14]. This aspect has become increasingly relevant in patient monitoring with the development of wearable PPG sensors attached to different body regions.

Recent advances in PPG signal processing have demonstrated that time-frequency analysis methods, particularly wavelet-based transforms, can significantly enhance noise robustness in PPG signal analysis. Wavelet-based motion artifact reduction algorithms have been demonstrated to enhance the visibility of pulse rate components and improve signal quality during movement, compared to common filtering methods [15]. In addition, deep learning and other machine learning approaches have been successfully applied to classify or reconstruct PPG signals in the presence of motion artifacts, improving heart rate estimation and enabling the extraction of cardiovascular parameters even in dynamic conditions [16]. Furthermore, machine learning, combined with spectral or time-frequency representations of PPG, has been used to estimate parameters such as pulse wave velocity and arterial stiffness, highlighting the potential of data-driven methods for extracting vascular biomarkers from noisy PPG recordings [17].

The current study investigates the effect of movement artifacts on the in-ear PPG signal induced by helicopter cabin vibrations. The PPG signal derives from the external ear canal by an innovative in-ear sensor approved as a medical device (c-med° alpha, described in Section 2.2.1). We hypothesize that high-frequency, low-amplitude movements of the helicopter seat induce artifacts in the PPG signal. Furthermore, we hypothesize that the artifacts resulting from this vibration impair vital sign extraction from the PPG signals based on the pulse contour.

## 2. Materials and Methods

### 2.1. Participants

This study is part of a multi-step study (Mountain Rescue Technology Study, (MoReTech; DRKS-ID: DRKS00025028) coordinated by the LMU Klinik, University Hospital Munich. Seventeen participants (mean age of 33.0; range 21–57 years, 5 female and 12 male) were recruited via announcement on social media. All participants underwent a standardized medical history-taking protocol and physical examination. Inclusion criteria for participation were (I) age between 18 and 80, (II) no history of diseases of the cardiocirculatory system, and (III) absence of clinical signs of inflammation of the ear canal. Each participant provided informed written consent before participation. All experimental procedures were approved by the local Ethics Committee (Ethical Committee N° 20-1136) and in agreement with the Declaration of Helsinki.

### 2.2. Instruments

#### 2.2.1. c-med° Alpha

**Figure 1:**
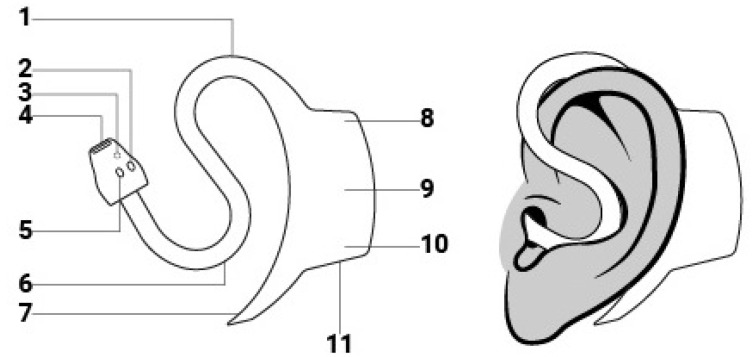
The c-med° alpha in-ear sensor unit consists of the following components: (1) sensor neck, (2) LED (red and infrared), (3) contact thermometer, (4) infrared thermometer, (5) photo diode, (6) anti tragus curve, (7) pickaxe, (8) status LED, (9) battery, (10) charging LED and (11) charging contacts.

The PPG signal measured in the external ear canal (PPG_ear_) was recorded using the commercially available medical in-ear sensor, the c-med° alpha (Cosinuss GmbH, Munich, Germany). Figure 1 shows a schematic representation of the device. The in-ear sensor contains two light-emitting diodes in the red and infrared spectrum (wavelength 655 nm and 940 nm) and a photodiode to detect the raw PPG_ear_ signal with a sampling rate of 200 Hz. Using the raw PPG_ear_ signal, the vital signs PR_ear_ and SpO_2ear_ get calculated every second. The recorded data is transmitted to a gateway via Bluetooth Low Energy (BLE 5.0) and is stored on the cosinuss° Health platform via the internet.

#### 2.2.2. Infinity™Delta

For recording the clinical reference data, a clinical standard monitoring system was used (Infinity™Delta, Draeger, Luebeck, Germany). It includes an electrocardiography with four limb electrodes for calculating the participant’s heart rate (HR_ref_). The QRS complexes in the electrocardiogram are identified, and the mean of 10 R-R intervals is calculated to determine the HR_ref_ after excluding the two longest and shortest intervals. The oxygen saturation (SpO_2ref_) is measured using the Masimo SET® with a finger clip at the left index finger and calculating the SpO_2__ref_ by mean of eight seconds.

#### 2.2.3. Ax6

The Ax6 (Axivity Ltd, Newcastle upon Tyne, UK) is a 6-axis movement sensor with a range of ±16 g. One sensor was placed at the headrest of the pilot seat, and one at the participant’s head with a head strap to detect the movements induced by the helicopter vibrations. The data was recorded with a sampling rate of 400 Hz.

### 2.3. Study Procedures

The study was performed at the Institute for Rotorcraft and Vertical Flight of the Technical University of Munich. The Rotorcraft Simulation Environment (ROSIE) consists of a realistic cockpit frame of a modified Bo105 fuselage and a 6-channel visual projection to create a realistic flight environment. To increase the realism of the ROSIE, a seat shaker, mounted to the pilot seat, generates realistic vibrations in the range of 5 to 200 Hz, depending on the simulated helicopter type and flight maneuver [18]. The participants could see a visual projection of the environment during the flight simulation from the pilot position.

After inserting the in-ear sensor in the participant’s right external ear canal and equipping the participant with the reference monitoring devices (ECG electrodes and finger clip for pulse oximetry), the participant was positioned in an upright-seated position at the pilot seat of the helicopter simulator. All participants were instructed not to talk or chew during the measurements and to keep their heads in contact with the headrest of the seat.

**Figure 2:**
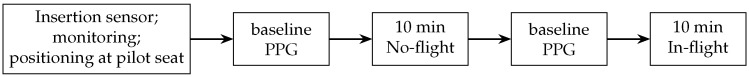
Steps of the experiment.

An adaptation for 2 min was performed without any exposure to movements to allow baseline PPG signals at the beginning of the experiment. The experiment was conducted in a fixed order: First, the patient remained upright seated without active movements and without cabin vibrations for 10 min (no-flight condition). After another 2 min adaptation phase, the consecutive in-flight condition was performed. The participant remained in the same position at the pilot seat, and the recorded flight was started. In the in-flight phase, the participant was exposed to the simulated cabin vibration by the seat shaker and could see the simulated environment on the visual projection. Figure 2 shows the different steps of the experiment.

**Figure 3:**
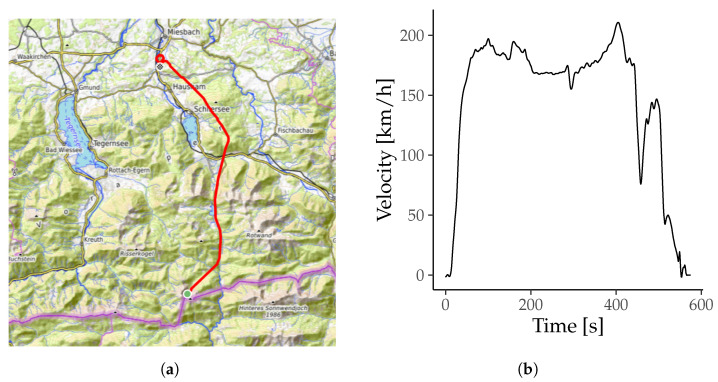
Track and velocity flown by the simulator for each patient. (**a**) The track of the simulated helicopter flight in bright red including start and endpoint marked by green and blue circles, drawn using gpx.studio with git hash 595ea8e (gpx.studio Team, 2025). (**b**) Velocity of the helicopter during flight (Indicated air speed).

The horizontal flight path and velocity are shown in Figure 3. The flight route started at a height of 1492 m ASL and descended in 576 s to the heliport at the hospital of Agatharied at 740 m ASL. The simulated helicopter is an H135 by Airbus Helicopters with four rotor blades and a main rotor speed of 7 Hz.

### 2.4. Data Analysis PPG_ear_

Seven signal quality indices (SQI) were calculated using the PPG_ear_ and resampled to 0.2 Hz by a moving average with a window size of five seconds. The calculation of the SQI is described below.

For statistical analysis, the mean of each SQI is calculated for each participant in the no-flight and the in-flight phase. It is then compared for each individual in the two flight phases in an intra-subject design. The normal distribution of the data was tested using the Shapiro–Wilk test. A paired *t*-test was used for parametric data to detect a statistically significant difference between the no-flight and in-flight phases, and a Wilcoxon signed-rank test was used for non-parametric data. A *p*-value < 0.05 was considered to be statistically significant. All descriptive data are represented using mean ± SD for parametric data and median and interquartile range for non-parametric data. Statistical analysis was performed using R 4.5.1 (R Core Team, 2025).

#### 2.4.1. Perfusion Index (PI)

The perfusion index is declared the gold standard for the quality of the PPG signal. It represents the amplitude of the alternating current component (AC) in relation to the direct current component (DC) [19].

(1) PI=AC/DC

#### 2.4.2. Skewness

Elgendi et al. investigated various signal quality indicators and their ability to distinguish between artifactually disturbed PPG signals [20]. They found that skewness could distinguish between undisturbed and disturbed PPG signals better than the gold standard PI [20].

(2) $$Skewness=\frac{1}{N}\sum_{i=1}^{N}{\left[x_{i}-\frac{{\hat{\mu }}_{x}}{\sigma }\right]}^{3}$$

The values ${\hat{\mu }}_{x}$ and σ are the estimated mean and standard deviation of the signal $x_{i}$ and *N* refers to the total number of the PPG samples [20].

#### 2.4.3. Entropy

In their study, Selvaraj et al. found that Entropy decreased significantly in PPG signals with motion artifacts [21]. The entropy serves as a quantitative indicator of the uncertainty in the signal by measuring how much its probability density function (PDF) diverges from a uniform distribution [20,21].

(3) $$Entropy=-\sum_{i=1}^{N}x_{i}^{\;2}\ln\left(x_{i}^{\;2}\right)$$

where *x* is the PPG signal and *N* is the number of data points [20].

#### 2.4.4. Kurtosis

Selvaraj et al. also found that kurtosis increased significantly in PPG signals affected by motion artifacts [21]. Kurtosis is a statistical metric that characterizes the shape of a data distribution relative to its mean, specifically indicating the presence of heavy or light tails and the degree of peakedness compared to a normal distribution [20,21].

(4) $$Kurtosis=\frac{1}{N}\sum_{i=1}^{N}{\left[x_{i}-\frac{{\hat{\mu }}_{x}}{\sigma }\right]}^{4}-3$$

The values ${\hat{\mu }}_{x}$ and σ represent the empirical mean and standard deviation of $x_{i}$, and *N* denotes the number of samples in the PPG signal [20].

#### 2.4.5. Omega

Omega is calculated from the PPG signal to determine blood oxygen saturation. The AC and DC ratios are calculated for the red and infrared wavelengths [1]. Omega is estimated by dividing the ratios of red and infrared wavelengths to calculate arterial oxygen saturation [1]. It is compared to an empirical calibration curve, determining the arterial oxygen saturation [1].

(5) $$Omega=\left(A_{ACR}/A_{DCR}\right)/\left(A_{ACIR}/A_{DCIR}\right)$$

#### 2.4.6. Quality Indicator (QI)

The in-ear sensor provides a signal quality indicator (QI, ranging from 0 to 100 (a.u.), with 100 being the most reliable) [22]. It is calculated at each PR data point and indicates reliability by measuring the PPG signal dominance concerning the perturbations [22].

#### 2.4.7. Valid Pulse Detection (VPD)

The in-ear sensor employs a valid pulse detection (VPD) algorithm, which analyzes pulse characteristics and similarities to accurately determine the validity of each pulse.

### 2.5. Data Analysis of Vital Signs

The vital signs SpO_2ear_ and PR_ear_ were calculated using the PPG_ear_ signal. The reference vital signs SpO_2ref_ and HR_ref_ were recorded manually every 30 s. For comparison, the in-ear data was resampled every 30 s, averaging over an interval of eighth seconds before the sample point.

The difference between the in-ear and reference vital signs was calculated for each time point and labeled as Bias. For detecting a statistically significant difference of the Bias_PR_ and Bias_Sp$O_{2}$_ between the no-flight and in-flight phase, the mean of the whole measurements from each participant in each flight phase was calculated and tested by the paired *t*-test for parametric data and the Wilcoxon signed-rank test for non-parametric data. The normal distribution of the data was tested using the Shapiro–Wilk test.

A Bland–Altman analysis with limits of agreement and the concordance correlation coefficient was calculated for the no-flight and in-flight phases to detect the accuracy of the in-ear sensor during both phases. Upper and lower limits of agreement were calculated with mean ± 1.96 SD. The strength of agreement between the vital sign_ear_ and vital sign_ref_ was defined as almost perfect, substantial, moderate, or poor to CCC values of >0.99; 0.95–0.99; 0.90–0.95 and <0.95, according to McBride et al. [23]. A *p*-value < 0.05 was considered to be statistically significant. All descriptive data are represented using mean ± SD.

## 3. Results

Initially, all 17 participants underwent all parts of the study. Due to the failed saving of the in-ear data from one participant, this participant’s data was excluded from further analysis. There are 16 participants with recorded data of 10 min of no-flight and 10 min of in-flight phase, totaling 320 min.

### 3.1. Motion Artifacts

**Figure 4:**
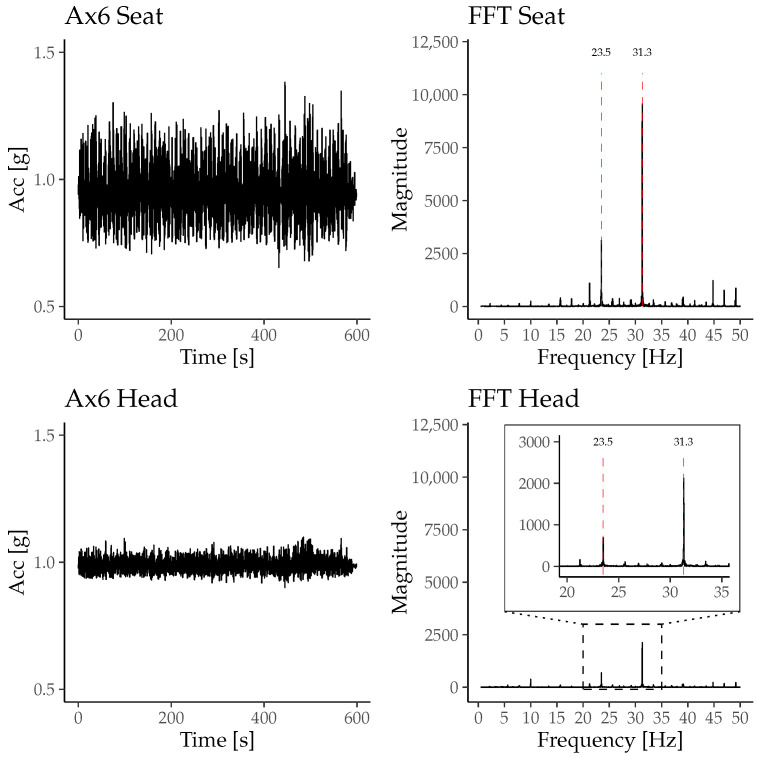
Absolute acceleration and corresponding FFT of the accelerometer signals measured by the Ax6 sensor at the pilot seat and at the participant’s head from participant 2’s data. The FFT plots reveal dominant peaks at approximately 23.5 Hz and 31.3 Hz, which correspond to the mechanical vibrations induced by the helicopter.

The vibration-induced mean acceleration in all participants during the in-flight phase was 0.97 ± 0.15 g for the Ax6 at the pilot seat and 0.98 ± 0.04 g for the Ax6 at the head strap. Figure 4 shows an example of the accelerometer data of participant 2, representing the data of the Ax6 located at the pilot seat and at the head strap. The fast Fourier transform (FFT) analysis of this data yielded two prominent peaks at 23.5 Hz and 31.3 Hz, as shown in Figure 4.

**Figure 5:**
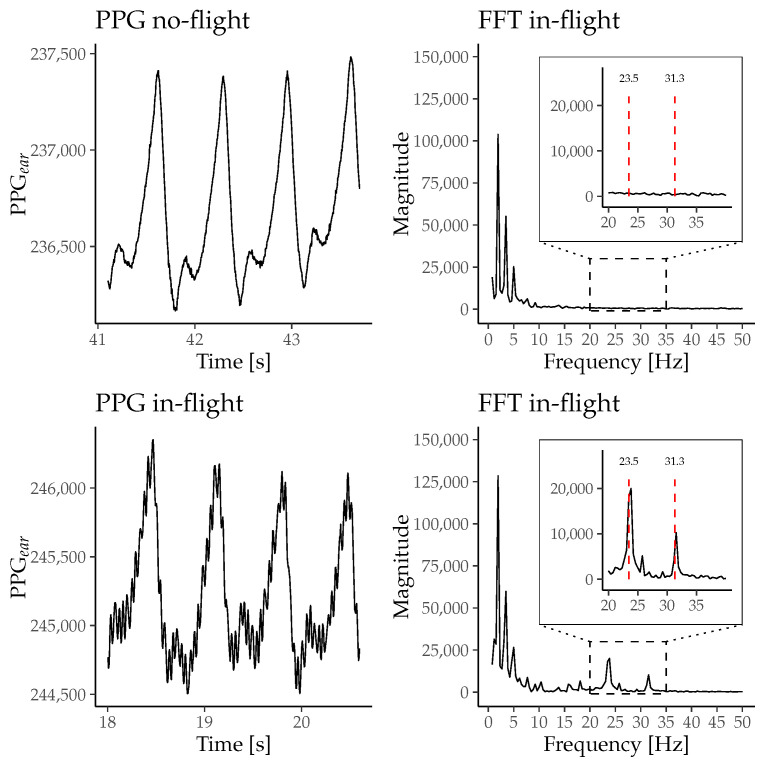
Representative segments of the PPG_ear_ signal recorded by the c-med° alpha from participant 1 during no-flight and in-flight phases, along with the corresponding FFT. The FFT during the in-flight phase shows additional peaks at 23.5 Hz and 31.3 Hz, which are absent in the no-flight phase. These peaks correspond to mechanical oscillations caused by helicopter vibrations.

For visualizing the effects of the vibrations on the PPG signal, Figure 5 shows the PPG_ear_ signal and the frequency distribution calculated with an FFT of participant 1 exemplarily during the no-flight and the in-flight phase. In addition, Figure 5 shows peaks at 23.5 Hz and 31.2 Hz during the in-flight phase in comparison to the no-flight phase.

### 3.2. PPG Signal Analysis

The Shapiro–Wilk test revealed a normal distribution for the difference of the means of all SQI, except Entropy IR, Entropy R, and Skewness R. Results are presented in Table 1. Table 2 shows the results of the paired *t*-test used for all SQI that did not have a significant Shapiro–Wilk test. Table 3 shows the results of the Wilcoxon signed-rank test that was used for Entropy IR, Entropy R, and Skewness R.

### 3.3. Vital Sign Analysis

On average, during the no-flight phase, the PR_ear_ was 69.4 ± 10.6 bpm, HR_ref_ 69.7 ± 11.6 bpm, and the Bias_PR_−0.3± 3.3 bpm. During the in-flight phase, the PR_ear_ was 69.9 ± 10.7 bpm, HR_ref_ was 70.4 ± 10.9 bpm, and the Bias_PR_ was −0.4 ± 2.9 bpm.

On average, during the no-flight phase the SpO_2ear_ was 96.8 ± 1.4%, SpO_2ref_ was 95.8 ± 1.4% and the Bias_Sp$O_{2}$_ was 1.0 ± 1.4%. During the in-flight phase the SpO_2ear_ was 97.2 ± 1.5%, SpO_2ref_ was 95.9 ± 1.4% and the Bias_Sp$O_{2}$_ was 1.3 ± 1.7%.

The Shapiro–Wilk test revealed a normal distribution for the Bias_Sp$O_{2}$_ (W = 0.97, *p*-value = 0.80) and a non-normal distribution for the Bias_PR_ (W = 0.71, *p*-value < 0.01). Therefore, a paired *t*-test was used to detect a statistically significant difference for the Bias_Sp$O_{2}$_ between no-flight and in-flight, and the Wilcoxon signed-rank test was used for the Bias_PR_.

There was no significant difference between the Bias in the no-flight phase and the in-flight phase, neither for the Bias_PR_ (z = 51, *p* = 0.39) nor the Bias_Sp$O_{2}$_ (t(30) = −0.47, *p* = 0.64).

**Figure 6:**
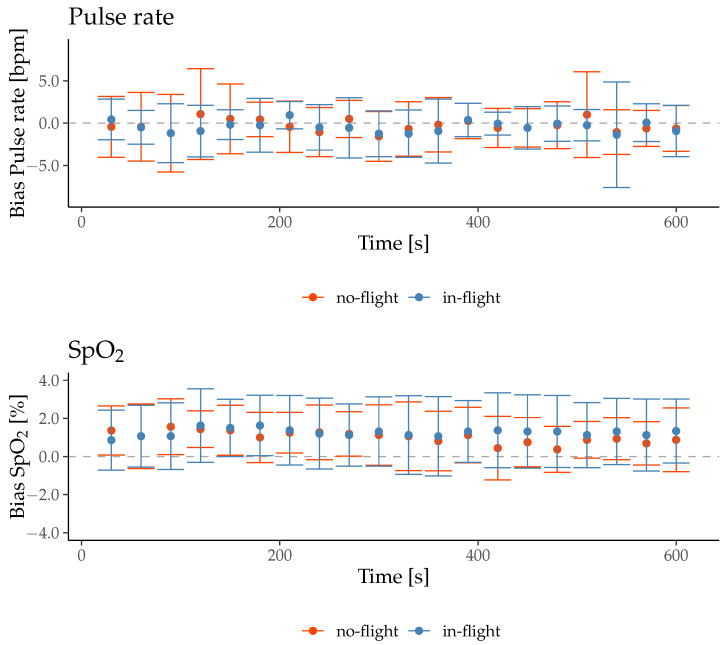
Mean ± SD of the Bias between in-ear vital signs and reference vital signs for no-flight and in-flight conditions.

The mean ± SD of the Bias at each time point is shown in Figure 6.

**Figure 7:**
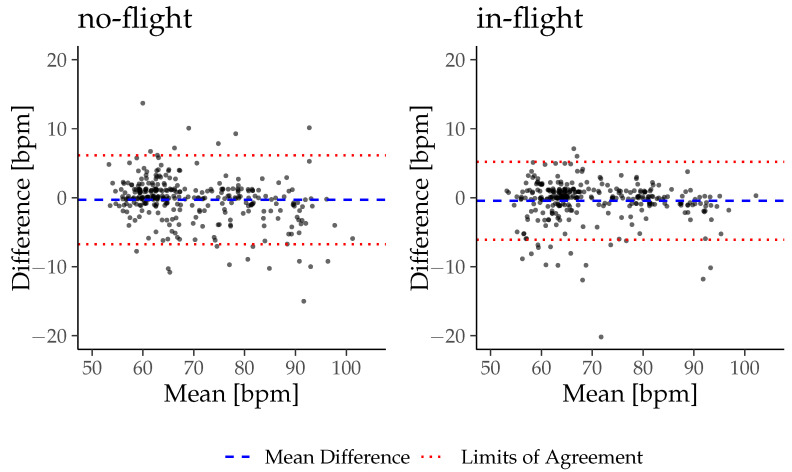
Bland-Altman plots for PR_ear_ in comparison to HR_ref_.

**Figure 8:**
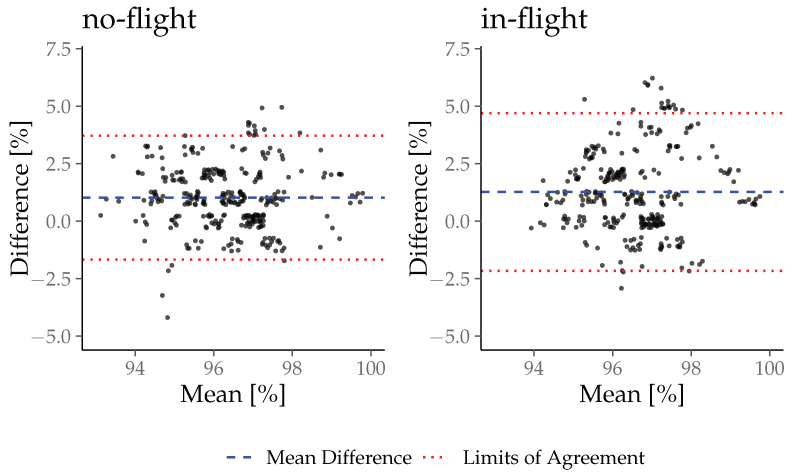
Bland-Altman plots for SpO_2ear_ in comparison to SpO_2ref_.

The results of the Bland–Altman analysis and the concordance correlation coefficient are presented in Table 4 with the Bland–Altman plots in Figure 7 and Figure 8. The c-med° alpha underestimates the PR by −0.30bpm during no-flight and −0.45 bpm during in-flight, while it overestimates the SpO_2_ by +1.02% during no-flight and +1.27% during in-flight on inter-subject average. The range of the limits of agreement (Upper LoA–Lower LoA) of the PR is wider during no-flight than during in-flight (8.42 bpm and 7.38 bpm), while it is narrower for SpO_2_ during no-flight than during in-flight (3.52% and 4.48%). The CCC indicates a substantial agreement between the PR_ear_ and the HR_ref_ during no-flight and in-flight (0.96 and 0.96), but a poor agreement between the SpO_2ear_ and the SpO_2ref_ during no-flight and in-flight (0.41 and 0.19).

## 4. Discussion

This study aimed to examine the influence of motion artifacts induced by helicopter cabin vibrations in PPG signals derived from the external ear canal. We developed insights into raw data alterations and their implications for extracting vital signs relevant to aeromedical patient monitoring. Our main findings are as follows:

(I) Helicopter cabin vibrations induced acceleration and movement in the upright seated persons’ heads during in-flight.
(II) These acceleration led to small amplitude, high frequency motion artifacts in the optical PPG raw signal derived from the external ear canal.
(III) The artifacts did not significantly influence quality indices of the PPG-based pulse contour obtained from the external ear canal, nor the extraction of vital sign parameters.

### 4.1. Cabin Vibrations and Associated Acceleration of Material and Participants

The primary source of helicopter cabin vibrations originates from the rotor system and the engines and propagates through the helicopter structure to the pilot seats [24]. During flight operations, persons in a seat are exposed to whole-body vibrations. The helicopter simulator used in this study did not comprise a rotor system. Instead, a seat shaker at the pilot seat generated vibrations comparable to actual flight vibrations induced by the rotor system [18]. Using the seat shaker, vibrations of a specific flight route were applied, resulting in distinct accelerations at the participant’s seat and head during the in-flight condition. The amplitude of the accelerations shows a consistent pattern slightly modulated by flight speed and the maneuvers of the simulated flight. The amplitudes are generally lower at patient’s head compared to the acceleration of the helicopter structure due to the dampening effects of the seat cushions and the human body. The acceleration of the participants’ heads were lower than those of the seat in all participants.

### 4.2. Motion Artifacts in the In-Ear PPG Signal

Studies applying PPG technology have investigated the physiological effects of body vibrations on different body functions (e.g., heart rate, microcirculation, and blood pressure) [25,26]. Herein, a systematic investigation of vibration-induced motion artifacts on the characteristics of the PPG raw signal is not yet available. The results of the present study indicate that vibrations of the head lead to acceleration and movement, thereby affecting the PPG_ear_ signal. Figure 5 shows that vibrations during in-flight led to a high-frequency, low-amplitude motion artifact. The frequency analysis of the optical PPG_ear_ signal reveals detectable additional frequency peaks during in-flight at 24 Hz and 31 Hz that directly correspond to the central frequencies of the acceleration patterns induced by the seat shaker at the pilot seat. However, this motion artifact on the PPG_ear_ signal did not significantly influence standard SQIs. In contrast to the current finding of non-impaired SQIs during vibrations, motion artifacts caused by physical activity significantly alter SQIs [27]. It could be hypothesized that motion during physical activity induces a higher amplitude of acceleration (with lower frequency) that may result in higher relative movements between the sensor units and their direct measuring sites. Therefore, distortion of the AC component or a wandering effect in the DC component are described [28]. In this study, motion artifacts were not caused by active or voluntary movement, but rather by whole-body exposure to simulated vibrations, which affected all potential sensor locations simultaneously. While common finger and earlobe PPG sensors are typically more susceptible to relative motion during physical activity, vibration-induced distortions may differ across anatomical sites due to differences in mechanical coupling and sensor fixation. In-ear PPG sensors may exhibit distinct responses to high-frequency, low-amplitude vibrations because of the confined anatomy of the external ear canal and the tight sensor–tissue contact. These factors distinguish vibration-related artifacts from conventional motion artifacts and should be considered when comparing PPG measurement sites. SQIs like the Perfusion Index, Skewness, and Omega mainly focus on contour characteristics of the pulsatile AC component of the PPG signal [1,19,20]. Those SQIs are unaffected by the in-flight vibration in our study, supporting the hypothesis that there is no influence on the vital signs extracted from the sensor data during in-flight phases. Spectral entropy is frequently used to assess the quality of long-term PPG measurements. Lower spectral entropy indicates a high-quality PPG signal time series with a peaked-power spectrum due to the oscillating nature of the pulse waves in the PPG signal [29]. Cabin vibrations during in-flight did not significantly change the entropy of the PPG signal in our study. The demonstrated consistency of spectral entropy can be interpreted as a preserved oscillatory stability of the PPG signal during in-flight conditions. The predominant frequencies in this signal may originate from the PR_ear_ with a potentially superimposed frequency domain at the harmonic frequency of the cabin vibration system. In our study, the latter does not deteriorate the overall predictability and quality of the pulsatile PPG_ear_ signal series. High Kurtosis values indicate clean and well-defined PPG_ear_ waveforms and are considered sensitive SQI for motion artifacts induced by physical activity [21]. Distortions of single waveforms in a time series of PPG pulse waves induce alterations in the tailored sections of the normal distribution, thus decreasing this SQI. While Kurtosis is highly sensitive for detecting artifacts, the in-flight cabin vibrations do not alter the metrics of the SQI in our study [21]. In summary, the overall reporting of unaffected SQIs during in-flight conditions supports the hypothesis that the high-frequency, low-amplitude cabin vibrations induce a characteristic noisy overlay on PPG signals. However, this motion artifact did not significantly change the PPG-derived pulse detection in our study.

### 4.3. Vital Signs

The recorded PPG signal is fundamental data for calculating the patient’s vital signs. Therefore, a good quality of the PPG_ear_ signal is essential for a valid vital sign extraction. An algorithm calculates the PR_ear_ using the PPG_ear_ signal as input and identifies the dominant frequency. Although a high-frequency harmonic PPG motion artifact is present during in-flight, the analysis for the PR showed a substantial agreement between the clinical reference measurement and the in-ear measurement during both the no-flight and in-flight phases. The algorithms in pulse oximeters for calculating the PR generally use filter techniques that exclude the frequencies outside the physiological range. It can be hypothesized that this filter hinders the motion artifacts at 23.5 Hz (=1410 bpm) and 31.3 Hz (=1878 bpm) from being extracted into PR, thereby generating robustness against these interfering artifacts. The results for analyzing the SpO_2_ agreement between the in-ear and reference device were less consistent than the PR. However, no significant difference was found in our study between the no-flight and in-flight phases. The algorithm for calculating SpO_2_ relies fundamentally on the differentiation between AC and DC components. Therefore, the delimitability of the AC and DC components and the differentiation of each pulse wave are more important than the exact pulse wave contour. The amplitude of the artifacts in our recordings is only a fraction of the amplitude of the AC signal. Therefore, we suspect this does not affect the distinguishability of the pulse curves and the separation of the AC and DC components. The results of the Bland-Altman analysis show a constant offset of the oxygen saturation between the in-ear and reference of +1.02 (no-flight) or +1.27 (in-flight). This continuous difference explains the poor agreement within the CCC. Interestingly, the limited agreement between SpO_2ear_ and SpO_2ref_ measurements did not differ between no-flight and in-flight phases, suggesting a systematic offset between measurement sites rather than an effect of vibration alone. Consistent with Davies et al., no-flight SpO_2ear_ values measured at the external ear canal tend to be slightly higher than those at the finger, likely reflecting anatomical and perfusion differences [30]. Importantly, in our study, the observed deviations remained within physiological limits and complied with the requirements of the relevant DIN standard [31]. This offset may also be influenced by sensor placement and signal coupling. Future studies could enhance the reliability of SpO_2ear_ by focusing on optimized sensor fixation, site-specific calibration, and multi-sensor integration to improve measurement robustness under challenging conditions.

The current study supports the robustness of PPG-based vital sign measurements derived from the external ear canal during simulated flight operations. The results should be interpreted considering some limitations in the study design and execution. The helicopter simulator uses seat shakers to generate vibrations at the cabin seats. Thus, generalizability for actual flight conditions is limited. In realistic flights, additional factors such as turbulence, variable noise, and complex seat movements may introduce artifacts not captured in our simulation. However, previous studies revealed similar characteristics and intensity of the vibrations with actual flights [18]. Future studies should validate in-ear PPG measurements under operational helicopter conditions to assess the full spectrum of vibration-induced effects on vital sign monitoring. The participants were measured in an upright position that might influence perfusion dynamics and even sensor adherence compared to supine lying persons. Further studies with different helicopter types and with supine-positioned participants are required. Due to limited infrastructure, the study could only be conducted with few participants. The relatively small sample size of this study may limit the generalizability of the results. Future investigations with larger cohorts are needed to confirm these findings and to enable more robust statistical analyses. All participants underwent the same study procedure, and the order of the study components was not randomized. During the in-flight phase, participants could perceive their surroundings from the pilot’s perspective, which can lead to motion sickness. This might indicate that the intraindividual analysis of vital signs during the two flight phases is not directly comparable. Thus, we focused on the intraindividual analysis of the difference between the in-ear and reference measurements in the vital sign analysis. Additionally, it should be noted that the study did not include a direct comparison of the PPG_ear_ signal with the finger-based PPG_ref_ signal under the same vibration conditions. Due to technical limitations, the PPG_ref_ signal could only be recorded via a conventional patient monitoring device, which provides processed vital parameters (SpO_2ref_ and PR_ref_) but does not allow access to the raw PPG signal. Therefore, our analysis focused specifically on comparing in-ear PPG signals between the no-flight and in-flight phases. While this approach enables the assessment of vibration-induced effects on in-ear PPG morphology and pulse extraction, it does not allow for the evaluation of site-specific differences in vibration susceptibility between the external ear canal and the finger. Future studies with simultaneous multi-site raw PPG recordings would be valuable to address this question. In summary, we did not find evidence to support our hypothesis that simulated helicopter vibrations significantly disturb the in-ear derived PPG signal. We could see an impact in the raw in-ear PPG signal, but no significant alteration in standard SQI or vital sign parameters.

## Figures and Tables

**Table 1:** Results of Shapiro–Wilk test PPG_ear_. * is used to mark significant results.

Parameter	W	*p*-Value
Omega	0.940	0.346
PI IR	0.945	0.419
Entropy IR	0.310	<0.001 *
Entropy R	0.310	<0.001 *
Kurtosis IR	0.960	0.667
Kurtosis R	0.963	0.720
Skewness IR	0.970	0.847
Skewness R	0.883	0.043 *
QI	0.929	0.232
VPD IR	0.670	0.587
VPD R	0.883	0.405

**Table 2:** Results of paired *t*-test PPG_ear_.

Parameter	Mean ± SD		df	t	*p*-Value
	No-Flight	In-Flight			
Omega	0.59 ± 0.06	0.57 ± 0.06	30	0.66	0.513
PI IR	1.03 ± 0.50	0.93 ± 0.49	30	0.55	0.587
Kurtosis IR	−0.56 ± 0.86	−0.55 ± 0.99	30	−0.04	0.969
Kurtosis R	−0.48 ± 1.13	−0.43 ± 1.37	30	−0.40	0.693
Skewness IR	0.21 ± 0.49	0.24 ± 0.46	30	−0.88	0.388
QI	68.65 ± 19.18	70.80 ± 17.73	30	−0.48	0.634
VPD IR	75.29 ± 36.62	79.38 ± 32.56	30	−0.57	0.573
VPD R	65.67 ± 41.01	67.26 ± 40.21	30	−0.18	0.861

**Table 3:** Results of Wilcoxon-signed rank test PPG_ear_.

Parameter	Mean ± SD		z	*p*-Value
	No-Flight	In-Flight		
Entropy IR	6.91 ± 0.10	6.91 ± 0.12	31.0	0.759
Entropy R	6.91 ± 0.10	6.91 ± 0.12	46.5	0.972
Skewness R	0.15 ± 0.58	0.19 ± 0.56	60.0	0.698

**Table 4:** Results of Bland–Altman analysis and CCC.

	PR [bpm]		SpO_2_ [%]	
	**No-Flight**	**In-Flight**	**No-Flight**	**In-Flight**
Difference	−0.30	−0.45	1.02	1.27
Lower LoA	−6.73	−6.10	−1.67	−2.16
Upper LoA	6.14	5.20	3.71	4.70
CCC	0.96	0.96	0.41	0.19

## Data Availability

The data presented in this study is not publicly available due to privacy and ethical restrictions. Anonymised data may be made available for research purposes from the corresponding author upon request.
